# Exosomal transfer of tumor-associated macrophage-derived hsa_circ_0001610 reduces radiosensitivity in endometrial cancer

**DOI:** 10.1038/s41419-021-04087-8

**Published:** 2021-08-30

**Authors:** Xiaobin Gu, Yonggang Shi, Meilian Dong, Li Jiang, Jing Yang, Zheyan Liu

**Affiliations:** Department of Radiation Oncology, The First Affiliated Hospital of Zhengzhou University, No. 1 Jianshe East Rd, Zhengzhou, 450000 Henan People’s Republic of China

**Keywords:** Cancer, Cell biology

## Abstract

The occurrence of radioresistance is a clinical obstacle to endometrial cancer (EC) treatment and induces tumor relapse. In this study, we found that tumor-associated macrophages (TAMs) enriched in EC specimens were determined to present an M2-like phenotype. In vitro, the coculture of M2-polarized macrophages significantly downregulated the radiosensitivity of EC cells by releasing exosomes. Hsa_circ_0001610 was found to be abundant in exosomes derived from M2-polarized macrophages (EXOs), and hsa_circ_0001610 knockdown eliminated the reduction effect of EXOs on the radiosensitivity of EC cells. The following mechanism research revealed that hsa_circ_0001610 functioned as the competing endogenous RNA of miR-139-5p, thereby upregulating cyclin B1 expression, which is a vital pusher of radioresistance in several types of cancer by regulating the cell cycle. Hsa_circ_0001610 overexpression reduced the radiosensitivity of EC cells, which was then reversed by miR-139-5p overexpression. In vivo, the promotion effect of EXOs on xenograft tumor growth in nude mice treated with irradiation was further reinforced after hsa_circ_0001610 overexpression. In conclusion, TAM-derived exosomes transferred hsa_circ_0001610 to EC cells, and the overexpressed hsa_circ_0001610 in EC cells released cyclin B1 expression through adsorbing miR-139-5p, thereby weakening the radiosensitivity of EC cells.

## Introduction

Endometrial cancer (EC) is the most frequent gynecologic malignancy, which seriously threatens the health of women worldwide [1, 2]. According to the current international guidelines, surgery combined with adjuvant therapy is the most established treatment option for patients with EC [3]. Radiotherapy, as one of the main adjuvant therapies for EC, successfully promotes the survival rate of patients with early-stage EC [4] and decreased loco-regional recurrence in patients with high-intermediate risk EC [5]. However, the occurrence of radioresistance is a clinical obstacle to EC treatment and induces tumor relapse [6]. Therefore, elucidating the mechanism of reduced radiosensitivity in EC is critical for enhancing radiotherapy potency.

Recently, the crucial role of the tumor microenvironment (TME) in tumor initiation and progression has been gradually realized. Tumor-associated macrophages (TAMs) are among the most abundant immune cells in the TME present at all stages during tumor progression. An evident TAM infiltration has been observed in human EC specimens compared with normal endometrial specimens, and the increased TAM number is positively correlated with the malignant progression of EC [7]. The research of Li et al. [8] determined that the TAMs in EC exhibited a polarized M2 phenotype. Also, Meng et al. [9] depleted TAMs in xenograft implants of melanoma cells in mice through local injection of macrophage-depleting liposomal clodronate and found that TAM depletion reinforced the antitumor effect of irradiation. These previous studies suggest that the accumulated TAMs are involved in the radioresistance of EC cells.

Increasing evidence shows that TAMs can modulate cancer progression via secreting exosomes (EXOs), a type of extracellular vesicles that mediates intercellular communication through transferring proteins, non-coding RNAs (ncRNAs), etc. from TAMs to tumor cells [10]. For instance, Feng et al. [11] reported that TAM-derived EXOs promoted intracranial aneurysm formation by delivering microRNA (miRNA)−155-5p to smooth muscle cells; Zheng et al. [12] found that TAM-derived exosomal miR-21 triggered cisplatin resistance in gastric cancer. However, whether TAM-derived EXOs could modulate the radiosensitivity of EC cells remains unclear.

Circular RNAs (circRNAs), a type of ncRNAs, have been proven to be a modulator of radiosensitivity in several cancer types, such as esophageal squamous cell carcinomas [13], nasopharyngeal carcinoma [14], and prostate cancer [15]. Utilizing RNA sequencing, Xu et al. [16] found that 209 circRNAs were abnormally expressed in the extracellular vesicles isolated from serum of patients with EC, suggesting that numerous circRNAs are packaged into EXOs and play a potential role in EC progression.

This study determined the role of TAM-derived EXOs in the radiosensitivity of EC cells and screen out the functional exosomal circRNA, which will provide new targets for enhancing the radiosensitivity of EC cells in clinical settings.

## Materials and methods

### Tissue specimens

A total of 25 EC specimens were obtained from patients who underwent surgical resection at The First Affiliated Hospital of Zhengzhou University. Twenty-five normal endometrial specimens obtained from patients with uterine fibroids undergoing hysterectomy were used as normal controls. No patients received preoperative chemotherapy or radiotherapy. All patients signed informed consent forms. This investigation involving human specimens was approved by the Ethics Committee of The First Affiliated Hospital of Zhengzhou University.

### Isolation of TAMs and tissue-resident macrophages (NTMs)

TAMs and NTMs were obtained from human EC specimens and normal endometrial specimens, respectively, as previously described [17]. Freshly human EC specimens and normal endometrial specimens were cut into small pieces and incubated with phosphate buffer containing 30 µl Liberase DL stock solution (28 U/ml; Roche, Switzerland), 120 µl Liberase TL stock solution (14 U/ml; Roche), and 30 µl DNase I (15 mg/ml; Sigma, USA) at 37 °C. Forty-five minutes later, the cell suspension was filtered using a 100 µm cell strainer and centrifuged for 5 min at 500 rcf. The cell pellet was resuspended in 3 ml red blood cell lysis buffer (Solarbio, China) to lyse red blood cells and centrifuged. Following this, the cell pellet was collected for qRT-PCR or resuspended in presort buffer (BD Biosciences, USA) at a density of 2 × 10^6^ cells/ml for flow cytometry.

### Generation of M2-polarized macrophages

M2-polarized macrophages were generated from human monocytic THP-1 cells as previously described [18]. THP-1 cells (Procell Life Science & Technology Co., Ltd., China) were incubated with phorbol 12-myristate 13-acetate (10 ng/ml; MedChemExpress, USA) for 24 h to differentiate into M0 macrophages. Then, M2-polarized macrophages were obtained by incubation with IL-4 (25 ng/ml; Thermo Fisher, USA) and IL-13 (25 ng/ml; Thermo Fisher) for another 48 h.

### Phenotypic analysis of TAMs, NTMs, and M2-polarized macrophages

Single-cell suspensions of TNMs and TAMs were prepared as described above and stained with BUV395 anti-human-CD45, APC anti-human CD11b, FITC anti-human-CD68, or BV-480 anti-human-CD206 (all antibodies were purchased from BD Biosciences). Then, labeled cells were analyzed on a FACSCanto II Flow Cytometer (BD Biosciences), and the data were processed using FlowJo Version 10.0. For the phenotypic analysis of THP-1-derived M2-polarized macrophages, cells were stained with APC anti-human-CD11b and FITC anti-human-CD206.

In addition, immunofluorescent staining was performed on TNMs and TAMs using anti-F4/80 and anti-CD206 antibodies (all purchased from Abcam, UK).

### Cell culture and transfection

Human EC cell lines Ishikawa and HEC-1B cells used in this study were purchased from Procell Life Science & Technology Co., Ltd (China). Ishikawa cells were cultured in Dulbecco’s modified Eagle’s medium (DMEM) containing 10% fetal bovine serum, and HEC-1B cells were cultured in Eagle’s minimum essential medium (EMEM) containing 10% fetal bovine serum in a 5% CO_2_ incubator at 37 °C.

The short hairpin RNA of circ_0001610 (sh-circRNA), miR-139-5p mimic, and their negative controls (shRNA and miR-NC) were synthesized by Guangzhou Ribobio Biotechnology Co., Ltd (China). Circ_0001610 overexpression vector (circ_0001610) and its negative control (vector) were synthesized by Guangzhou Geneseed Biotechnology Co., Ltd (China). For cell transfection, Ishikawa/ HEC-1B cells were seeded in six-well plates at a density of 1 × 10^6^ cells/well. When cells reached 65% confluence, sh-circRNA/shRNA/circ_0001610/vector was transfected into cells using Lipofectamine™ 3000 (Thermo Fisher, USA), and miR-139-5p mimic/ miR-NC was transfected into cells using riboFECT™ CP Reagent (Guangzhou Ribobio Biotechnology Co., Ltd. China).

### EXOs extraction and identification

The EXOs were isolated from the supernatant of M2-polarized macrophages cultured for 48 h in EXO-free culture medium. The extraction process was conducted as previously described [19]. Briefly, the supernatant was centrifuged at 2000 × *g* for 10 min and 10,000 × *g* for ½ h to remove dead cells and cell debris, followed by centrifugation at 110,000 × *g*. Then, the cell pellets were resuspended in PBS and centrifuged at 110,000 × *g* to obtain the EXOs.

The isolated EXOs were identified by transmission electron microscope (TEM) and the Western blot. For TEM, isolated EXOs were fixed with 1% glutaraldehyde, adsorbed onto a formvar/carbon-coated grid, and negatively stained with uranyl acetate solution. Then, EXOs were visualized under TEM (Hitachi, Japan).

EXO^shRNA^, EXO^sh-circRNA^, EXO^vector^, and EXO^circ_0001610^ were extracted from the culture medium of M2-polarized macrophages transfected with shRNA, sh-circRNA, vector, and circ_0001610 by the above method, respectively.

### Cell viability assay

Ishikawa or HEC-1B cells were treated according to the corresponding protocols and then seeded in 96-well plates. Twenty-four hours later, cells were treated with 4 Gy irradiation at a dose of 0.5 Gy/min (Varian2300EX, Varian, USA). Seven-two hours later, cells in each well were incubated with 10 µl 3-(4,5-dimethylthiazol-2-yl)-2,5-diphenyltetrazolium bromide (5 mg/ml; Beyotime Biotechnology, China) for 4 h and 100 µl Fromazan for another 4 h. Then, the absorbance of each well at 570 nm was examined using a microplate reader.

### Cell proliferation assay

Cell proliferation was measured by the colony formation assay. Ishikawa or HEC-1B cells were treated according to the corresponding protocols and then seeded in six-well plates at a density of 1000 cells/well. Twenty-four hours later, cells were treated with 4 Gy irradiation and then cultured under a normal condition. Two weeks later, the plates were fixed with methanol and then stained with Giemsa (Beyotime Biotechnology, China). Colonies with ≥50 cells were counted using an inverted microscope (Olympus Life Science, Japan).

### Cell apoptosis assay

The Annexin V-FITC Apoptosis Detection Kit (Beyotime Biotechnology, China) was employed to assess cell apoptosis. Ishikawa or HEC-1B cells were treated according to the corresponding protocols and then seeded in six-well plates. Twenty-four hours later, cells were treated with 4 Gy irradiation and then cultured under a normal condition for another 72 h. Subsequently, cells were resuspended in Annexin V-FITC binding buffer at a density of 1 × 10^6^ cells/ml and incubated with 5 µl Annexin V-FITC and 10 µl propidium iodide (PI) in the dark for 20 min. Cell apoptosis was assessed by a FACSCanto II Flow Cytometer (BD Biosciences).

### Cell invasion assay

Cell invasion was measured by the Transwell assay. Ishikawa or HEC-1B cells were treated according to the corresponding protocols, and then 2 × 10^4^ cells were seeded in the upper chamber pre-coated with Matrigel Matrix (Corning, USA). Also, 600 µl DMEM/EMEM containing 10% FBS was added into the lower chamber. Cells that invaded the reverse side of the membrane were fixed and stained using crystal violet (Beyotime Biotechnology) after 48 h. An inverted microscope (Olympus Life Science) was used to photograph the stained cells.

### Cell cycle assay

The cell cycle was assessed using flow cytometry. Ishikawa or HEC-1B cells were treated according to the corresponding protocols and resuspended in phosphate buffer. After centrifuging, the cell pellets were fixed with ethanol overnight. On the second day, cells were stained with a staining solution containing PI (Thermo Fisher, USA) and RNAse A (Thermo Fisher) for 30 min. Samples were analyzed by a FACSCanto II Flow Cytometer (BD Biosciences).

### RNA-fluorescence in situ hybridization (RNA-FISH)

RNA-FISH was performed to examine the subcellular localization of hsa_circ_0001610 in EC cells. Ishikawa or HEC-1B cells were seeded on the coverslips. The next day, the coverslips were incubated with 4% paraformaldehyde and 0.5% Triton X-100, followed by the hybridization solution containing Cy3-labeled hsa_circ_0001610 probe (produced by Guangzhou Ribo Biotechnology Co., Ltd). After counterstaining with DAPI, the coverslips were photographed using a confocal microscope.

### Dual-luciferase reporter assay

The interplay between hsa_circ_0001610 and miR-139-5p was assessed by dual-luciferase reporter assay. The sequence of hsa_circ_0001610 was subcloned into the pmirGLO-basic plasmid (Promega, USA) (circ_0001610). The mutant sequence of hsa_circ_0001610 (the mutant sides were located at the potential binding sites of hsa_circ_0001610 and miR-139-5p) was subcloned into the pmirGLO-basic plasmid (Mutant). Then, the well-grown HEK-293T cells were co-transfected with circ_0001610/Mutant and miR-NC/miR-139-5p mimic. Two days after transfection, the relative luciferase activities of Mutant and circ_0001610 were examined using the dual-luciferase reporter assay system (Promega).

To determine the interplay between miR-139-5p and *CCNB1*, the CCNB1 3′-untranslated region wild type (CCNB1 3′-UTR WT; containing the sequence of *CCNB1* 3′-UTR) and CCNB1 3′-UTR mutant (containing the mutant sequence of *CCNB1* 3′-UTR and the mutant sides were located at the potential binding sites of miR-139-5p and *CCNB1*) were prepared. The relative luciferase activity of CCNB1 3′-UTR WT/CCNB1 3′-UTR mutant in HEK-293T cells transfected with miR-NC/miR-139-5p mimic was measured as described above.

### Western blot

Ishikawa, HEC-1B cells were collected, and the protein samples were extracted using RIPA lysis buffer (Shanghai Absin Biological Technology Co., Ltd, China). The expression level of cyclin B1 in protein samples was measured by Western blot as previously described [20]. To identify the isolated EXOs, protein samples were extracted from EXOs using the Exosome Protein Extraction Kit (Shanghai Hifun Biotechnology Co., Ltd. China). Then, the expression levels of CD63, CD9, and CD81 were measured by Western blot. To identify the epithelial-to-mesenchymal transition (EMT) in the tumor xenograft model mice, the protein samples were extracted from the xenograft tumor tissues, and the protein levels of zinc finger E-box binding homeobox 1 (ZEB1), N-cadherin, β-catenin, vimentin, and E-cadherin were measured. The primary antibodies used in this study were as follows: anti-cyclin B1 antibody (1:50,000), anti-CD63 antibody (1:1000), anti-CD9 antibody (1:2000), anti-CD81 antibody (1:1000), anti-ZEB1 (1:500), N-cadherin (1 µg/ml), β-catenin (1:5000), vimentin (1:2000), and E-cadherin (1:10,000). All antibodies were purchased from Abcam.

### RNA pull-down assay

The RNA pull-down assay was employed to further confirm the binding between hsa_circ_0001610 and miR-139-5p. Guangzhou Ribobio Biotechnology Co., Ltd (China) synthesized the biotinylated miR-139-5p (Biotin-miR-139-5p) and its negative control (biotinylated control RNA, referred to as control RNA). Ishikawa and HEC-1B cells were lysed using NP40 lysis buffer (Boster Biological Technology Co., Ltd, China), followed by incubation of 20 nM Biotin-miR-139-5p or control RNA. The biotin-coupled RNA complex was pulled down by incubating with streptavidin-coated magnetic beads (Thermo Fisher). The enrichment of hsa_circ_0001610 in the bound fractions was examined using qRT-PCR.

### qRT-PCR

Total RNA samples were isolated from TAMs, NTMs, Ishikawa cells, HEC-1B cells, and xenotransplanted tumors using Trozol (Thermo Fisher). TaqMan MicroRNA assays (Thermo Fisher) were employed to quantify the expression levels of miR-139-5p, with U6 serving as an internal control. For the quantification of mRNA and circRNA, PrimeScript RT Master Mix was used to synthesize cDNA samples. Then, qRT-PCR was performed using 100 ng cDNA samples and SYBR Premix Ex Taq II (Takara, Japan) with GAPDH serving as an internal control. The expression levels of mRNA, miRNA, and circRNAs were figured out by the 2^−ΔΔCT^ method.

### Tumor xenografts in nude mice

Before the in vivo study, the EXOs were isolated from the culture medium of normally cultured M2-polarized macrophages, EXOs that overexpressed hsa_circ_0001610 (EXO^circ_0001610^) and negative control of EXO^circ_0001610^ (EXO^Vector^) were isolated from the culture medium of M2-polarized macrophages transfected with circ_0001610 and vector, and EXOs that silenced hsa_circ_0001610 (EXO^sh-circRNA^) were isolated from the culture medium of M2-polarized macrophages transfected with sh-circRNA, respectively. Also, 5 × 10^6^ Ishikawa or HEC-1B cells were suspended in a 50 µl mixture of DMEM and Matrigel (volume ratio = 1:1) and subcutaneously injected into the BALB/c nude mice (4-week-old; Beijing Charles River Experimental Animal Technology Co., Ltd, China). When tumors grew to 100 mm^3^, mice were allocated to five groups: Control (*n* = 6), EXO (*n* = 6), EXO^circ_0001610^ (*n* = 6), EXO^Vector^ (*n* = 6), and EXO^sh-circRNA^ (*n* = 6). In the control group, the tumors were locally irradiated with 10 × 2.5 Gy for 5 days. In the EXO/EXO^Vector^/EXO^circ_0001610^/ EXO^sh-circRNA^ group, 10 µg EXO/EXO^Vector^/EXO^circ_0001610^/EXO^sh-circRNA^ was injected into the center of the tumor every 2 days, and the tumors were locally irradiated with 10 × 2.5 Gy for 5 days. Tumor volumes were measured every week after the beginning of local irradiation. Four weeks after the beginning of local irradiation, mice were sacrificed and the xenotransplanted tumors were collected. All animal experiments were approved by the Committee on the Ethics of The First Affiliated Hospital of Zhengzhou University.

### Immunohistochemical staining

Tumor tissues were prepared into 4-µm-thick sections, followed by dewaxing and re-watering. Then, sections were immersed in 3% hydrogen peroxide. Ten minutes later, sections were orderly incubated with a blocking solution and anti-cyclin B1/Ki-67 antibody (Abcam). On the next day, sections were reacted with a secondary antibody and then counterstained with hematoxylin. A BX63 fluorescence microscope (Olympus Life Science) was used to photograph the stained sections.

### Statistical analysis

GraphPad Prism 7.0 (GraphPad, USA) was used to perform statistical analyses. The results are expressed as the means ± standard deviations. The statistical significance of the differences between the two experimental groups was evaluated using a Student’s *t* test, and *p* < 0.05 was considered statistically significant.

## Results

### Macrophages are enriched in the cancerous tissues of patients with EC and characterized by the M2-polarized phenotype

**Fig. 1:**
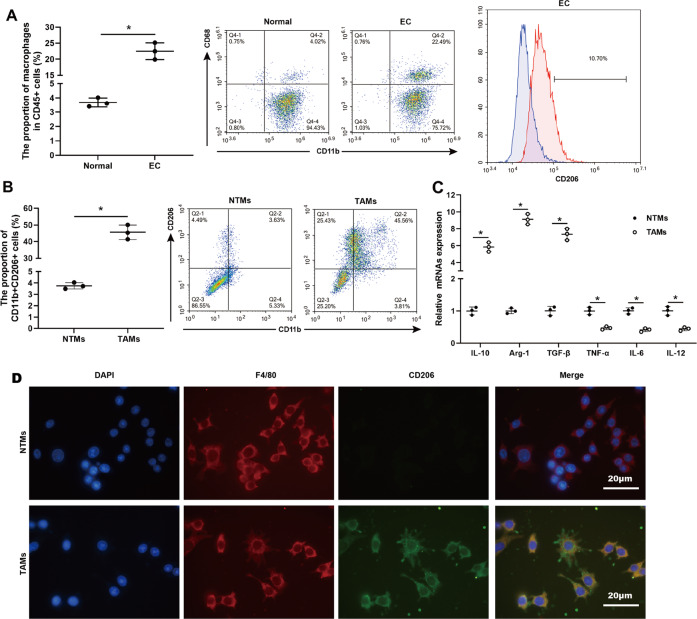
Accumulation of M2-like TAMs in cancerous tissues of patients with EC. **A** Flow cytometry analysis and quantification of macrophages in human EC specimens (*n* = 3) and normal endometrial specimens (*n* = 3). Left: CD45^+^ cells were gated and then analyzed the CD11b^+^ CD68^+^ cells. Right: The proportion of CD206^+^ cells in human EC specimens. **B** Flow cytometry analysis and quantification of CD11b^+^ CD206^+^ macrophages in tissue-resident macrophages (NTMs, *n* = 3) and tumor-associated macrophages (TAMs, *n* = 3). **C** qRT-PCR analysis of IL-10, arginase-1 (Arg-1), transforming growth factor-beta (TGF-β), tumor necrosis factor-alpha (TNF-α), IL-6, and IL-12 mRNA levels in NTMs (*n* = 3) and TAMs (*n* = 3). *p < 0.05. **D** The representative images of immunofluorescent staining for F4/80 (red) and CD206 (green) performed on TAMs and NTMs. Nuclei were counterstained with DAPI (blue). Scale bar = 20 μm.

To evaluate the distribution of macrophages in the human EC specimens, the expressions of CD11b and CD68, two macrophage markers [21], were measured. Flow cytometric quantification showed a higher density of CD11b^+^ CD68^+^ macrophages in CD45^+^ immune cells in EC specimens than in normal endometrial specimens (Fig. 1A). Through analyzing the expression of CD206, a specific surface marker of M2-polarized macrophages [22], we found that the proportion of CD206^+^ cells in EC specimens was 10.7%, but the proportion of CD206^+^ cells in normal endometrial specimens was too low to be detected (Fig. 1A). TAMs in EC specimens were verified as an M2-like phenotype, characterized by an increased proportion of CD11b^+^ CD206^+^ cells (Fig. 1B). Meanwhile, elevations in mRNA levels of M2 markers (IL-10, Arg-1, and TGF-β [23]) and reductions in mRNA levels of M1 markers (TNF-α, IL-6, and IL-12 [24]) were observed in TAMs in EC specimens compared with NTMs in normal endometrial specimens (Fig. 1C), further confirming that the TAMs in EC specimens were primarily macrophage subpopulation with an M2 phenotype. The results of immunofluorescent staining also demonstrated that TAMs in EC specimens were F4/80 positive and CD206 positive while NTMs in normal endometrial specimens were F4/80 positive and CD206 negative (Fig. 1D).

### M2-polarized macrophage-derived EXOs accounted for weakening the radiosensitivity of EC cells

**Fig. 2:**
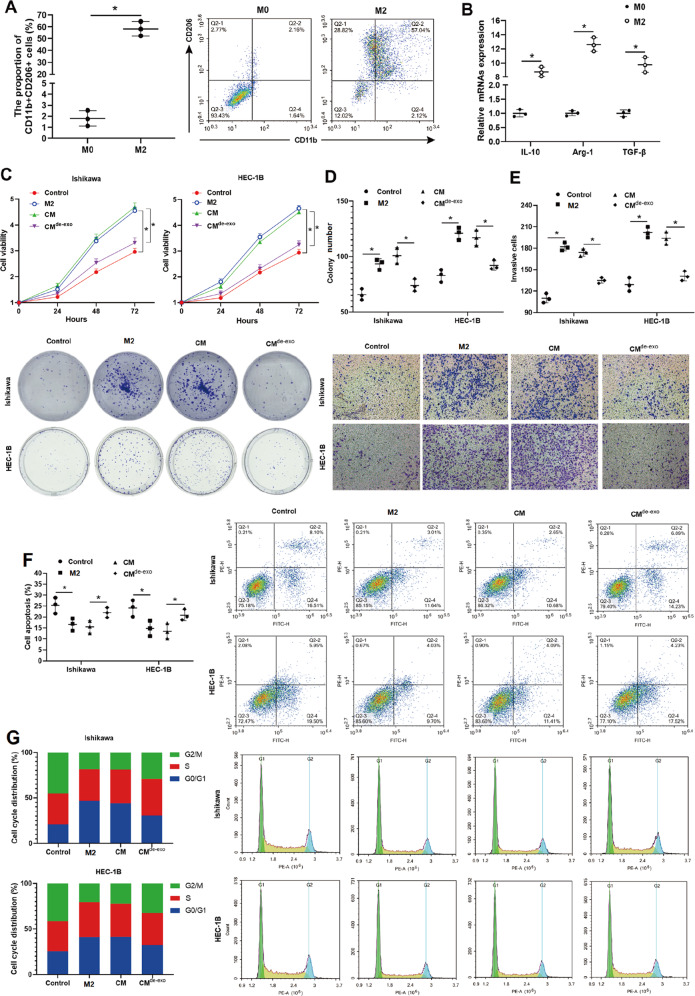
M2-polarized macrophage-derived exosomes weakened the radiosensitivity of EC cells. **A** Flow cytometry analysis and quantification of CD11b^+^ CD206^+^ cells and **B** qRT-PCR analysis of IL-10, Arg-1, and TGF-β mRNA levels were performed on nonpolarized M0/M2-polarized macrophages generated from THP-1 cells. **C**–**G** The conditioned medium (CM) and conditioned medium deleted exosomes (CM^de-EXO^) were harvested from the M2-polarized macrophages culture medium. EC cell lines (Ishikawa and HEC-1B) were monocultured or cocultured with M2-polarized macrophages/CM/CM^de-EXO^ and then treated with 4 Gy irradiation. **C** Cell viability was measured by 3-(4,5-dimethylthiazol-2-yl)-2,5-diphenyltetrazolium bromide (MTT) assay. **D** Cell proliferation was measured by colony formation assay. **E** Cell invasion was measured by Transwell assay. **F** Cell apoptosis was measured by flow cytometry. **G** Cell cycle distribution was determined by propidium iodide (PI) staining. *p < 0.05.

To assess the functional biology of TAMs on EC cells in vitro, M2-polarized macrophages were generated from THP-1 cells, and the influence of M2-polarized macrophages on EC cells’ viability, proliferation, invasion, and radiosensitivity was examined. As shown in Fig. 2A, M2-polarized macrophages expressed higher expression levels of CD11b and CD206. The mRNA levels of IL-10, Arg-1, and TGF-β in M2-polarized macrophages were also upregulated compared to those in nonpolarized M0 macrophages (Fig. 2B). Compared with monocultured Ishikawa and Ishikawa cells cocultured with M0 macrophages, Ishikawa cocultured with M2-polarized macrophages exhibited higher viability, increased colony numbers, and enhanced invasive ability (Supplementary Fig. 1A–C), indicating that M2-polarized macrophages promoted the malignant phenotype of EC cells.

Further, the effect of TAMs on the radiosensitivity of EC cells was evaluated. Following treatment with 4 Gy irradiation, EC cell lines (Ishikawa and HEC-1B) cocultured with M2-polarized macrophages presented higher cell viability, increased colony numbers, reinforced invasive ability, and decreased cell apoptosis compared with monocultured EC cell lines (Fig. 2C–F) and EC cell line cocultured with M0 macrophages (Supplementary Fig. 1D–F). Meanwhile, the cell cycle distribution also showed that the rate of G2/M-arrested cells was lessened in EC cell lines (Ishikawa and HEC-1B) cocultured with M2-polarized macrophages than in monocultured EC cell lines after 4 Gy irradiation treatment (Fig. 2G). These data suggested that M2-polarized macrophages weakened the radiosensitivity of EC cells.

Given that emerging evidence confirms that EXOs derived from TAMs play a central role in the communication between tumor cells and TAMs [25, 26], we then explored whether TAM-derived EXOs mediated the radiosensitivity of EC cells. The conditioned medium (CM) and conditioned medium deleted EXO (CM^de-EXO^) were harvested from the M2-polarized macrophage culture medium (Supplementary Fig. 2). Like M2-polarized macrophages, the CM treatment also upregulated cell viability (Fig. 2C), proliferation (Fig. 2D), and invasion (Fig. 2E), and downregulated cell apoptosis (Fig. 2F) and G2/M cell cycle arrest (Fig. 2G) in EC cell lines (Ishikawa and HEC-1B) exposed to 4 Gy irradiation. In contrast, EC cell lines (Ishikawa and HEC-1B) incubated with CM^de-EXO^ presented lower cell viability, suppressed capacities of proliferation and invasion, promoted cell apoptosis, and induced G2/M cell cycle arrest compared with EC cell lines incubated with CM after 4 Gy irradiation treatment (Fig. 2C–G). Collectively, the above data indicated that EXOs derived from M2-polarized macrophages attenuated the radiosensitivity of EC cells.

### Exosomal hsa_circ_0001610 mediated the weakening effect of M2-polarized macrophage-derived EXOs on the radiosensitivity of EC cells

**Fig. 3:**
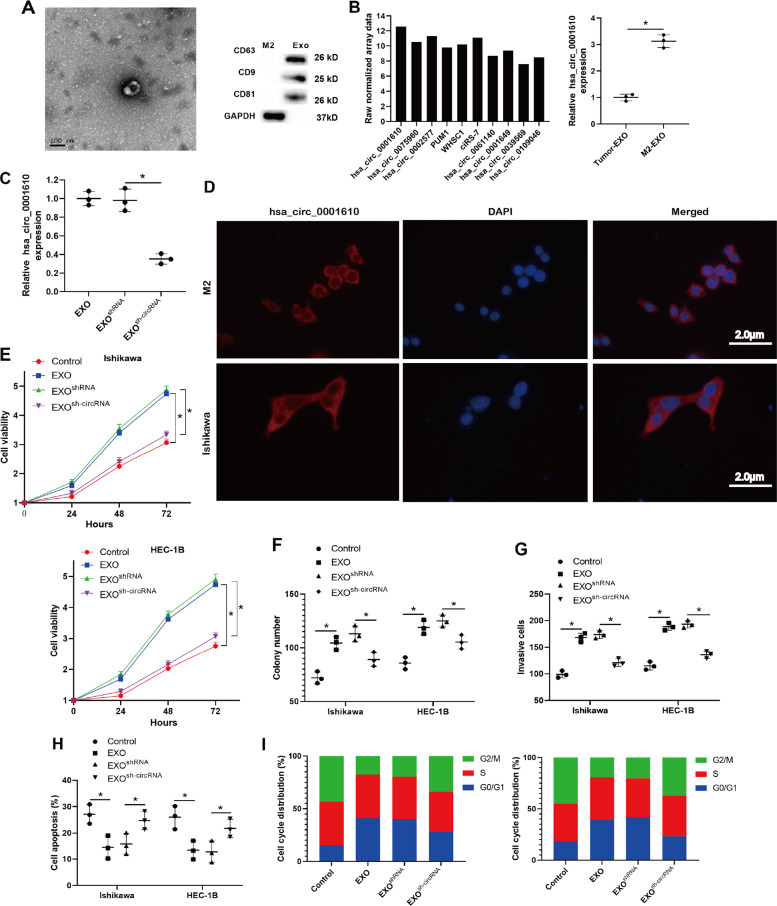
Exosomal hsa_circ_0001610 mediated the weakening effect of M2-polarized macrophage-derived EXOs on the radiosensitivity of EC cells. **A** Representative transmission electron microscopy image of M2-polarized macrophage-derived EXOs (Scale bar = 100 nm). **B** Left: the expression profiles of ten candidate circRNAs in M2-polarized macrophage-derived EXOs were generated by a circRNA microarray analysis. Right: the expression level of hsa_circ_0001610 in EC cell-derived EXOs and M2-polarized macrophage-derived EXOs was measured by qRT-PCR. **C** The expression level of hsa_circ_0001610 in M2-polarized macrophage-derived EXOs (EXO), EXOs that silenced hsa_circ_0001610 (EXO^sh-circRNA^), and the negative control of EXO^sh-circRNA^ (EXO^sh-RNA^) was measured by qRT-PCR. **D** The cellular localization of hsa_circ_0001610 in M2-polarized macrophages and Ishikawa cells (Scale bar = 2.0 μm). **E**–**I** EC cell lines (Ishikawa and HEC-1B) were normally cultured (Control) or treated with EXO/EXO^sh-RNA^/EXO^sh-circRNA^, and then treated with 4 Gy irradiation. Cell **E** viability, **F** proliferation, **G** invasion, **H** apoptosis, and **I** cell cycle were measured. *p < 0.05.

Following, the mechanism by which EXOs derived from M2-polarized macrophages conferred radioresistance of EC cells was investigated. The EXOs were isolated from M2-polarized macrophages by differential centrifugation. TEM imaging analysis showed that purified EXOs morphologically resembled a cup-shaped structure and were around 100 nm in size (Fig. 3A). Western blot analysis showed that purified EXOs were positive for the EXO-specific markers CD63, CD9, and CD81 [27] (Fig. 3A). These data confirmed the success of EXO isolation. Increasing evidence reports that several circRNAs participate in EC progression and that circRNAs encapsulated in EXOs play a vital role in implementing the cellular function of EXOs [28, 29]. To explore whether circRNAs were involved in M2-polarized macrophage-derived EXOs-mediated radiosensitivity of EC cells, the following study was conducted. Through a circRNA microarray analysis, we obtained the expression profiles of 10 candidate circRNAs reported to be related to EC development [16, 30–36] in M2-polarized macrophage-derived EXOs and found that hsa_circ_0001610 was the most abundant (Fig. 3B). The following results of qRT-PCR also confirmed that hsa_circ_0001610 was abundant in M2-polarized macrophage-derived EXOs rather than EC cell-derived EXOs (Fig. 3B). The RNA-FISH assay showed that hsa_circ_0001610 was localized to the cytoplasm of M2-polarized macrophages and Ishikawa cells (Fig. 3D).

Then, EXOs that silenced hsa_circ_0001610 (EXO^sh-circRNA^), its negative control (EXO^shRNA^), and normal EXOs were prepared (Fig. 3C) and added into the culture mediums of Ishikawa and HEC-1B cells before 4 Gy irradiation treatment. Compared with monocultured EC cell lines, EC cell lines treated with EXOs presented increased cell viability (Fig. 3E), promoted cell proliferation (Fig. 3F), enhanced cell invasion (Fig. 3G), suppressed cell apoptosis (Fig. 3H), and lessened rate of G2/M-arrested cells (Fig. 3I) after 4 Gy irradiation treatment. However, the EXO-induced radioresistance of EC cells was partially reversed when silencing hsa_circ_0001610 in M2-polarized macrophage-derived EXOs. As shown in Fig. 3E–I, EXO^sh-circRNA^, rather than EXO^sh-RNA^, efficiently reduced cell viability, repressed cell proliferation, weakened cell invasion, reinforced cell apoptosis, and augmented the rate of G2/M-arrested cells in EC cell lines treated with 4 Gy irradiation. Overall, these data suggest that M2-polarized macrophage-derived EXOs reduce the radiosensitivity of EC cells by loading hsa_circ_0001610.

### The interplay between hsa_circ_0001610, miR-139-5p, and cyclin B1

**Fig. 4:**
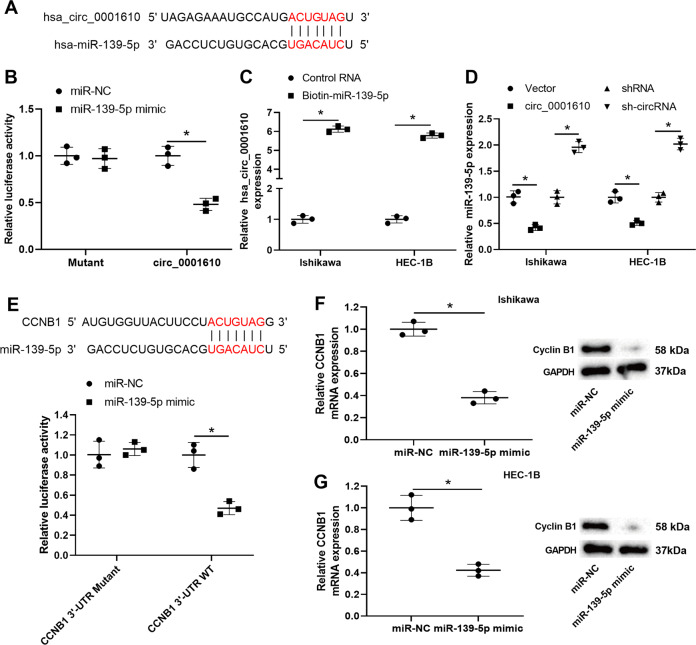
The interplay between hsa_circ_0001610, microRNA (miR)-139-5p, and cyclin B1. **A** The potential binding sites between hsa_circ_0001610 and miR-139-5p. **B** Dual-luciferase reporter assay using HEK-293T cells co-transfected with pmirGLO-circ_0001610 vector/pmirGLO-mutant-circ_0001610 vector and miR-139-5p mimic/the negative control of miR-139-5p mimic (miR-NC). **C** qRT-PCR analysis of hsa_circ_0001610 level in the streptavidin captured fractions from the Ishikawa and HEC-1B cell lysates after incubation with biotinylated miR-139-5p (biotin-miR-139-5p) or control RNA. **D** qRT-PCR analysis of miR-139-5p in the Ishikawa and HEC-1B cells after transfection with circ_0001610/the negative control of circ_0001610 (vector)/sh-circRNA/shRNA. **E** Upper: the potential binding sites between *CCNB1* (the encoding gene of cyclin B1) and miR-139-5p. Below: dual-luciferase reporter assay using HEK-293T cells co-transfected with pmirGLO-CCNB1 3′-UTR mutant vector/ pmirGLO-CCNB1 3′-UTR wild type (WT) vector and miR-139-5p mimic/ miR-NC. The mRNA and protein levels of cyclin B1 were measured in **F** Ishikawa and **G** HEC-1B cells after transfection with miR-139-5p mimic/miR-NC. *p < 0.05.

As reported, circRNAs could act as competing endogenous RNAs (ceRNAs), competitively combining with miRNAs to affect the expression levels of target mRNAs, thereby exerting their modulatory effect on radiosensitivity in several types of cancers [13, 14]. According to the bioinformatics database (Circular RNA Interactome), 55 miRNAs were found to have potential binding sites to hsa_circ_0001610. Of them, 14 miRNA have been reported to be related to EC development (Supplementary Fig. 3). Their expression levels were measured using qRT-PCR in Ishikawa cells transfected with hsa_circ_0001610. As shown in Supplementary Fig. 3, miR-139-5p level demonstrated the most significant change upon hsa_circ_0001610 overexpression. Therefore, miR-139-5p was selected for the following investigation. The potential binding sites between miR-139-5p and hsa_circ_0001610 were shown in Fig. 4A. The results of the dual-luciferase reporter assay showed that the relative luciferase activity of the pmirGLO-circ_0001610 vector, rather than the pmirGLO-mutant-circ_0001610 vector, was significantly decreased by miR-139-5p mimic transfection in HEK-293T cells (Fig. 4B). The results of the RNA pull-down assay showed a sixfold enrichment of hsa_circ_0001610 in the biotin-miR-139-5p-captured fraction compared with the control RNA-captured fraction in EC cell lines (Fig. 4C). Meanwhile, miR-139-5p expression was downregulated by hsa_circ_0001610 overexpression whereas upregulated by hsa_circ-0001610 knockdown in EC cell lines (Fig. 4D). These data confirmed that hsa_circ_0001610 was directly bound to miR-139-5p and negatively regulated its expression in EC cell lines.

Using a bioinformatics database (TargetScan), we found that cyclin B1, a vital pusher of radioresistance in several types of cancer by regulating the cell cycle [37–39], was the potential target of miR-139-5p and the potential binding sites between *CCNB1* (the encoding gene of cyclin B1) and miR-139-5p are shown in Fig. 4E. A distinct reduction in the relative luciferase activity of pmirGLO-CCNB1 3′-UTR WT vector in miR-139-5p mimic-transfected HEK-293T cells indicated the interplay between miR-139-5p and *CCNB1* (Fig. 4E). Furthermore, the results of qRT-PCR and Western blot demonstrated that both mRNA and protein levels of cyclin B1 were lessened by miR-139-5p mimic transfection in EC cell lines (Fig. 4F, G), which suggested that cyclin B1 expression was negatively regulated by miR-139-5p.

### Hsa_circ_0001610 weakened the radiosensitivity of EC cells by suppressing miR-139-5p expression

**Fig. 5:**
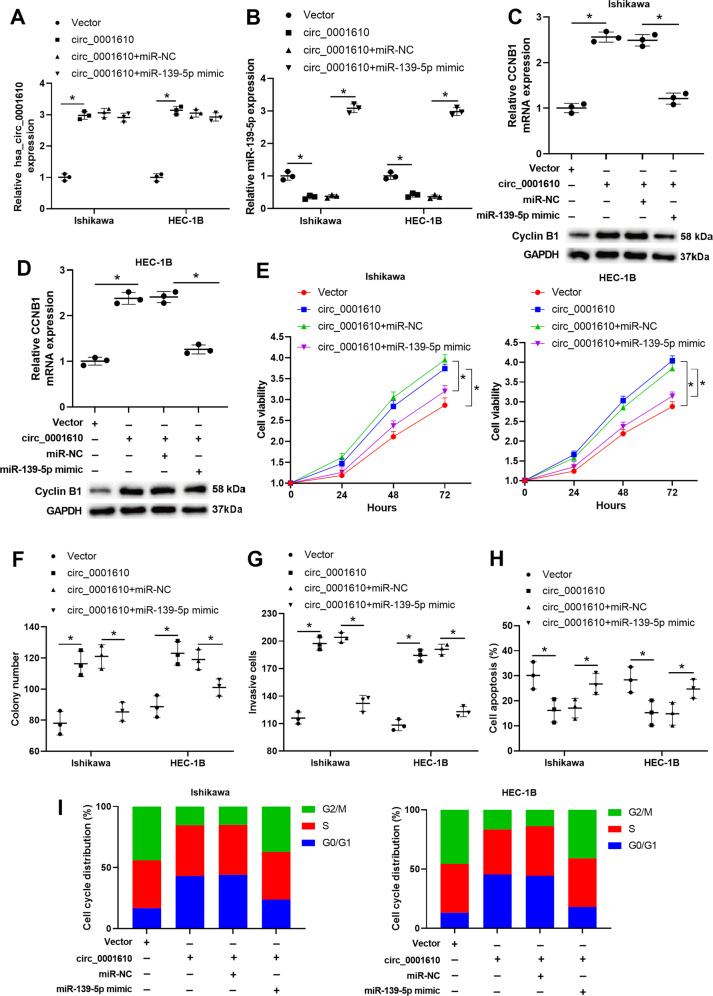
Hsa_circ_0001610 weakened the radiosensitivity of EC cells by suppressing the miR-139-5p expression. Ishikawa and HEC-1B cells were transfected with vector, hsa_circ_0001610, hsa_circ_0001610 + miR-NC, or hsa_circ_0001610 + miR-139-5p mimic, followed by exposure to 4 Gy irradiation. The expression levels of **A** hsa_circ_0001610 and **B** miR-139-5p was measured by qRT-PCR. The mRNA and protein levels of cyclin B1 in **C** Ishikawa and **D** HEC-1B cells were measured by qRT-PCR and Western blot, respectively. **E** Cell viability was measured by MTT assay. **F** Cell proliferation was measured by colony formation assay. **G** Cell invasion was measured by Transwell assay. **H** Cell apoptosis was measured by flow cytometry. **I** Cell cycle distribution was determined by PI staining. *p < 0.05.

Next, we explored whether hsa_circ_0001610 reduced the radiosensitivity of EC cells by modulating miR-139-5p. Ishikawa and HEC-1B cells were transfected with vector, hsa_circ_0001610, hsa_circ_0001610+miR-NC, or hsa_circ_0001610+miR-139-5p mimic, followed by exposure to 4 Gy irradiation. The hsa_circ_0001610 transfection overtly promoted circ_0001610 level (Fig. 5A), decreased miR-139-5p level (Fig. 5B), and increased cyclin B1 level (Fig. 5C, D) in EC cell lines treated with 4 Gy irradiation. The miR-139-5p mimic transfection efficiently reversed the regulatory effect of hsa_circ_0001610 on the expressions of miR-139-5p and cyclin B1, which was manifested in upregulating miR-139-5p level (Fig. 5B) and reducing cyclin B1 level (Fig. 5C, D) in EC cell lines treated with 4 Gy irradiation + hsa_circ_0001610 + miR-139-5p mimic. Furthermore, in EC cell lines treated with 4 Gy irradiation, hsa_circ_0001610 overexpression elevated cell viability (Fig. 5E), boosted cell proliferation (Fig. 5F), enhanced cell invasion (Fig. 5G), suppressed cell apoptosis (Fig. 5H), and reduced G2/M cell cycle arrest (Fig. 5I). In contrast, miR-139-5p overexpression re-enhanced the radiosensitivity of EC cells in the presence of hsa_circ_0001610, which was manifested in the downregulations of cell viability (Fig. 5E), proliferation (Fig. 5F), invasion (Fig. 5G), the upregulations of cell apoptosis (Fig. 5H), and the rate of G2/M-arrested cells (Fig. 5I) in EC cell lines treated with 4 Gy irradiation + hsa_circ_0001610 + miR-139-5p mimic.

### Exosomal hsa_circ_0001610 weakened the radiosensitivity of EC cells in vivo

**Fig. 6:**
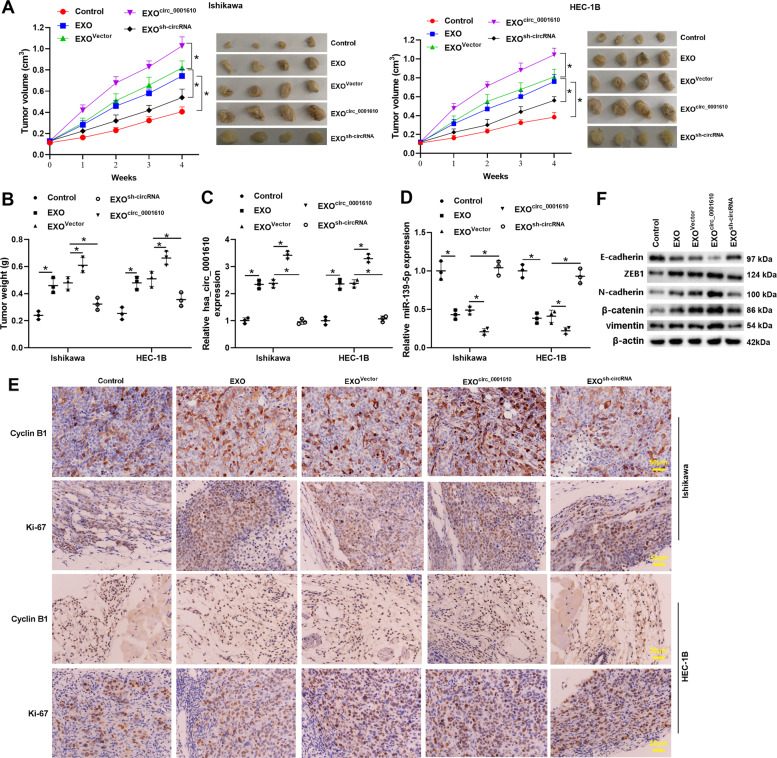
Exosomal hsa_circ_0001610 weakened the radiosensitivity of EC cells in vivo. Ishikawa or HEC-1B cells were subcutaneously injected into the nude mice to establish the tumor xenograft model. When tumors grew to 100 mm^3^, mice were treated with irradiation (Control group; *n* = 6), irradiation + EXO (EXO group; *n* = 6), irradiation + EXOs that overexpressed hsa_circ_0001610 (EXO^circ_0001610^; *n* = 6), irradiation + the negative control of EXO^circ_0001610^ (EXO^vector^ group; *n* = 6), and irradiation + EXOs that silenced hsa_circ_0001610 (EXO^sh-circRNA^; *n* = 6). **A** The tumor volumes were measured every week after the beginning of local irradiation. Four weeks after the beginning of local irradiation, mice were sacrificed, the xenotransplanted tumors were collected and photographed. **B** Weight of tumors. The expression levels of **C** hsa_circ_0001610 and **D** miR-139-5p in tumors were measured. **E** Representative images of immunohistochemical staining for cyclin B1 and Ki-67 (Scale bar =  50 μm). **F** The protein levels of E-cadherin, zinc finger E-box binding homeobox 1 (ZEB1), N-cadherin, β-catenin, and vimentin. *p < 0.05.

Then, the role of exosomal hsa_circ_0001610 in the radiosensitivity of EC cells was investigated in vivo. Ishikawa or HEC-1B cells were subcutaneously injected into nude mice to establish the tumor xenograft model. When tumors grew to 100 mm^3^, mice were treated with irradiation, irradiation + EXO, irradiation + EXO^vector^, irradiation + EXO^circ_0001610^, or irradiation + EXO^sh-circRNA^. The expression level of hsa_circ_0001610 in the tumors, measured by qRT-PCR, confirmed that the injection of EXO/EXO^vector^/EXO^circ_0001610^ successfully transferred hsa_circ_0001610 to the xenotransplanted tumors of nude mice, among which the mice that received EXO^circ_0001610^ injection presented the most abundant hsa_circ_0001610 (Fig. 6C). As shown in Fig. 6A, B, the tumor volume and tumor weight of nude mice injected with EC cells + EXO was increased compared with nude mice only injected with EC cells. In addition, the expression level of miR-139-5p was decreased (Fig. 6D), and the protein levels of cyclin B1 and Ki-67 (a proliferation marker [40]) (Fig. 6E) were promoted in the tumors of nude mice injected with EC cells + EXO compared to the tumors of nude mice injected with EC cells. However, the effects of EXO on tumor volume, tumor weight, and the expression levels of miR-139-5p, cyclin B1, and Ki-67 were removed when silencing hsa_circ_0001610 (EXO^sh-circRNA^) (Fig. 6A–D). Compared with EXO^vector^, the administration of EXO^circ_0001610^ resulted in increased tumor volume and weight (Fig. 6A, B), a lower miR-139-5p level (Fig. 6D), and higher protein levels of cyclin B1 and Ki-67 (Fig. 6E) in the tumors of nude mice injected with EC cells. Moreover, EXO facilitated the EMT in the tumor xenograft model mice after irradiation, manifesting as the upregulations of ZEB1, N-cadherin, β-catenin, and vimentin, and the downregulation of E-cadherin. The effect of EXO on EMT was enhanced by circ_0001610 overexpression (EXO^circ_0001610^) while removed by circ_0001610 knockdown (EXO^sh-circRNA^). These data suggested that exosomal hsa_circ_0001610 contributed to the EXO-weakened radiosensitivity of EC cells.

## Discussion

In recent years, researchers have come to appreciate that tumors comprise not only tumor cells but also TME. TAMs are the vital components of TME, which promote tumor formation, development, recurrence, and metastasis [41, 42]. Radiotherapy triggers cell death by inducing double-stranded DNA damage. However, under radiotherapy, TAMs promote the radioresistance of cells by inducing immunosuppression, ultimately leading to radiotherapy failure [43, 44]. Therefore, targeting TAMs may be a novel direction for enhancing radiotherapy potency. Here, we identified a mechanism by which TAMs communicate with EC cells to reduce their radiosensitivity. Our data demonstrated that TAM-derived EXOs transferred hsa_circ_0001610 to EC cells, and the overexpressed hsa_circ_0001610 in EC cells released cyclin B1 expression through adsorbing miR-139-5p, thereby weakening the radiosensitivity of EC cells.

Macrophages are extremely plastic cells classified as either M1 or M2 phenotypes [45]. M1-polarized macrophages participate in innate host defense and exhibit an antitumor effect, while M2-polarized macrophages participate in humoral immunity and exhibit a pro-tumor effect [46]. Increasing evidence confirms that TAMs are characterized by an M2-polarized phenotype in several types of solid cancer, such as breast cancer [47], gastric cancer [48], and lung cancer [49]. In the present study, an increased TAMs infiltration was observed in the EC specimens relative to normal endometrial specimens, and the following phenotypic analysis revealed that TAMs in EC specimens presented an M2-like phenotype (Fig. 1). Consistent with our data, the research of Gu et al. [7] also reported that TAMs in patients with EC tended to contain a polarized M2 phenotype. Meanwhile, TAMs are proposed to enhance cancer cell resistance against radiotherapy. Shiao et al. [50] depleted macrophages in the xenograft mammary tumor of mice through colony-stimulating factor 1 (CSF-1) antibody and found that macrophage depletion apparently suppressed tumor growth followed by radiotherapy. Xu et al. [51] reported that compared with irradiation treatment alone, a combination of irradiation and CSF-1 receptor kinase inhibitor more efficiently retard tumor growth in a mouse model of prostate cancer by limiting TAMs recruitment. In line with the previous study, compared with the monocultured EC cell lines, EC cell lines cocultured with M2-polarized macrophages presented higher cell viability after irradiation treatment, indicating that M2-polarized macrophages decreased the radiosensitivity of EC cells (Fig. 2). What’s more, we obtained CM and CM^de-EXO^ from the M2-polarized macrophage culture medium and added them into the culture medium of EC cell lines. The results showed that, like M2-polarized macrophages, CM also promoted the viability of EC cell lines treated with irradiation, whereas this ability was removed in the absence of EXOs (Fig. 2), suggesting that M2-polarized macrophages decreased the radiosensitivity of EC cells by secreting EXOs.

As reported, EXOs play a crucial role in cancer progression by transferring various bioactive molecules, such as circRNAs, between cancer cells and TME [52]. Through circRNA microarray and qRT-PCR analysis, we found that hsa_circ_0001610 was abundant in M2-polarized macrophage-derived EXOs (Fig. 3). The experiments that followed showed that knockdown hsa_circ_0001610 in M2-polarized macrophage-derived EXOs removed the promoting effect of M2-polarized macrophage-derived EXOs on the viability of EC cell lines treated with irradiation, suggesting that exosomal hsa_circ_0001610 reduced the radiosensitivity of EC cells (Fig. 3). This finding was also verified by the accelerative tumor growth observed in the EXO^circ_0001610^ + irradiation-treated xenograft mouse model (Fig. 6). In the previous study, Ye et al. [36] found the upregulation of hsa_circ_0001610 in the EC tissues of women with grade 3 EC compared with normal endometrial specimens utilizing circRNA sequencing, suggesting the potential role of hsa_circ_0001610 in EC for the first time. Compared to the previous study, the present study not only confirmed the promoting effect of hsa_circ_0001610 on the radioresistance of EC cells but also clarified that the high hsa_circ_0001610 expression in EC tissues may dependent on TAM-derived EXOs transport. In addition, we analyzed the association between hsa_circ_0001610 and the overall survival of EC patients we enrolled and found there was no significant correlation between them (*p* = 0.77, Supplementary Fig. 4). Due to the limitation of small sample size, the results in this study may not sufficient to confirm the correlation between hsa_circ_0001610 and the overall survival of EC patients. In future work, we will enroll more EC patients with or without radioresistance to further explore the potential role of hsa_circ_0001610 in prognosis and diagnosis in EC patients with or without radioresistance.

Radiotherapy causes cell apoptosis by damaging DNA double strands and suppressing cell cycle checkpoint activation [53]. G2/M phase has been reported to be the most radio-sensitive stage of the cell cycle. Several chemotherapeutic agents, such as celecoxib and docetaxel, exert their radiosensitizing effect on tumor cells by inducing G2/M phase arrest [54, 55]. In the present study, an obvious upregulation of G2/M cell cycle arrest was observed in EC cells treated with EXO^sh-circRNA^ (Fig. 3), hinting that the modulatory effect of hsa_circ_0001610 on the radiosensitivity of EC cells may rely on the cell cycle. Cyclins are critical regulators of the cell cycle. As a key mitotic cyclin, cyclin B1 induces the G2/M phase transition of the cell cycle, thus reducing the radiosensitivity in several types of cancers, such as nasopharyngeal carcinoma, squamous cell carcinoma, and non-small cell lung cancer [56–58]. Through suppressing cyclin B1 expression, proteasome activator complex subunit 3 (PSME3) triggers cell cycle arrest at the G2/M phase and enhances the radiosensitivity of colorectal cancer cells [37]. In EC, the cyclin B1 is overexpressed, and its high expression is closely related to the increased proliferative potential of EC cells [59]. However, whether cyclin B1 contributes to the radioresistance in EC is still unknown. In the present study, using Dual-luciferase reporter assay and RNA pull-down assay, we determined that cyclin B1 was the downstream target of hsa_circ_0001610/miR-139-5p axis and that hsa_circ_0001610 increased cyclin B1 expression via functioning as the ceRNA of miR-139-5p (Figs. 4 and 5). In EC cell lines treated with 4 Gy irradiation, hsa_circ_0001610 overexpression upregulated cyclin B1 expression, reinforced cell viability, and reduced the G2/M cell cycle arrest (Fig. 5). However, the miR-139-5p mimic abrogated the promotion effect of hsa_circ_0001610 overexpression on cyclin B1, reduced cell viability, and augmented G2/M cell cycle arrest in circ_0001610 + irradiation-treated EC cell lines (Fig. 5). These data suggest that the weakening effect of circ_0001610 overexpression on radiosensitivity of EC cells is related to its modulatory effect on cyclin B1. The bioinformatics database TargetScan predicted numerous targets of miR-139-5p, and the reason we chose cyclin B1 for this study is that our study focuses on the role of the hsa_circ_0001610/miR-139-5p axis on cell cycle regulation. In the following studies, we will continue to validate other targets of miR-139-5p to more comprehensively elucidate the role of hsa_circ_0001610/miR-139-5p in the progression of EC.

In summary, our study demonstrates that TAM-derived EXOs function as carriers of hsa_circ_0001610, transferring hsa_circ_0001610 to EC cells to adsorb miR-139-5p and release the expression of cyclin B1, thereby weakening the radiosensitivity of EC cells. However, due to objective limitations, we were unable to collect enough samples of EC patients with radioresistance to verify the correlation of this mechanism in clinical samples. Although there are some shortcomings, the present study identifies a new mechanism by which TAMs communicate with EC cells to reduce their radiosensitivity and indicates hsa_circ_0001610 as a potential intervention target of radioresistance in EC.

## Supplementary information

Supplementary Figure 1

Supplementary Figure 2

Supplementary Figure 3

Supplementary Figure 4

## Data Availability

The datasets used and/or analyzed during the current study are available from the corresponding author on reasonable request.
