# Supplementary material for: Exosomal transfer of tumor-associated macrophage-derived hsa_circ_0001610 reduces radiosensitivity in endometrial cancer
Source: Cell Death Dis. 2021 Aug 30;12(9):818. doi: 10.1038/s41419-021-04087-8 (PMC8405633; doi:10.1038/s41419-021-04087-8)
Supplement: Supplementary file 1 — Supplementary Figure 1 [file 41419_2021_4087_MOESM1_ESM.docx]

**
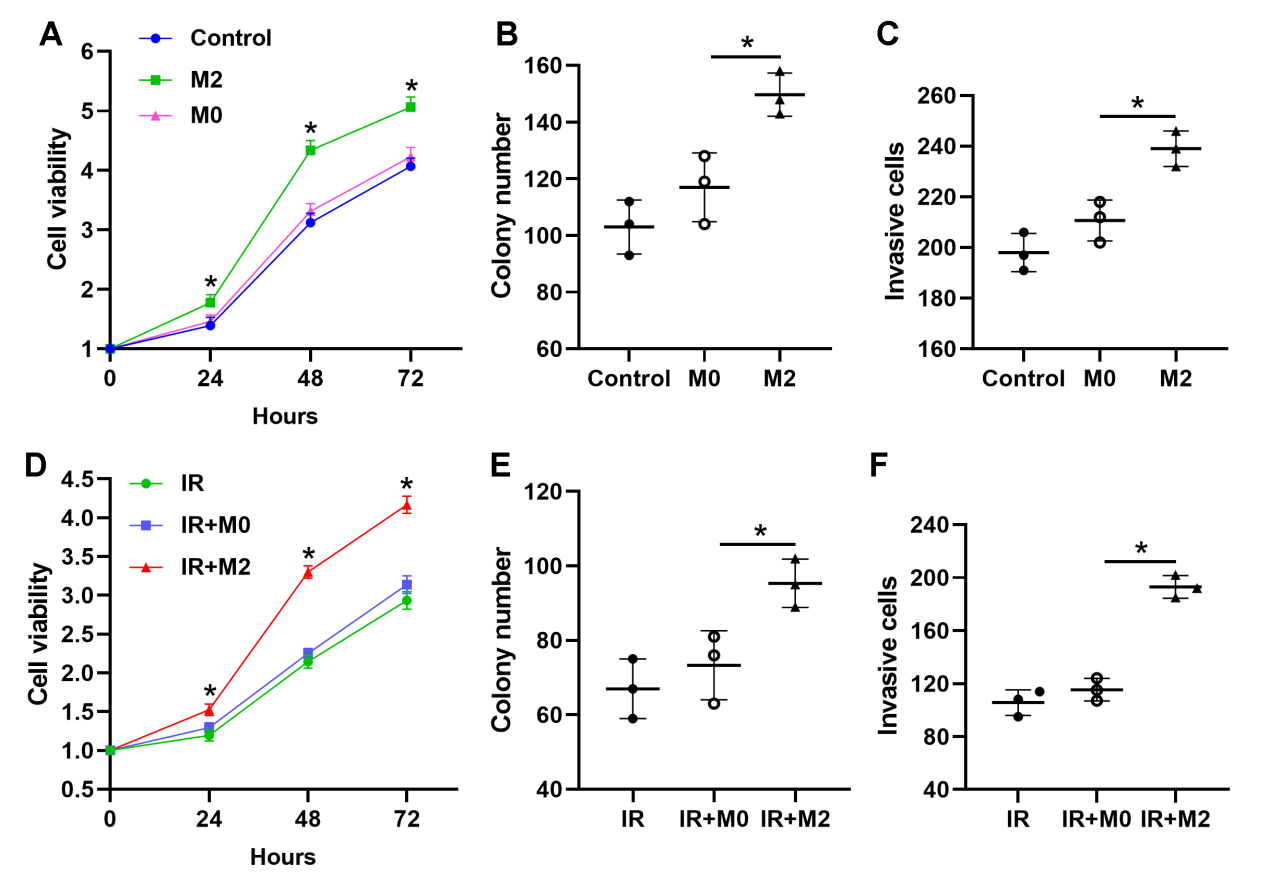
Supplementary Figure 1** Cell viability, colony formation, and cell invasion were measured in human EC cells (Ishikawa) monocultured or cocultured with M2-polarized macrophages without (A-C) or with (D-E) irradiation treatment. **P*<0.05 vs monocultured Ishikawa cells.
