# Supplementary material for: Exosomal transfer of tumor-associated macrophage-derived hsa_circ_0001610 reduces radiosensitivity in endometrial cancer
Source: Cell Death Dis. 2021 Aug 30;12(9):818. doi: 10.1038/s41419-021-04087-8 (PMC8405633; doi:10.1038/s41419-021-04087-8)
Supplement: Supplementary file 3 — Supplementary Figure 3 [file 41419_2021_4087_MOESM3_ESM.docx]

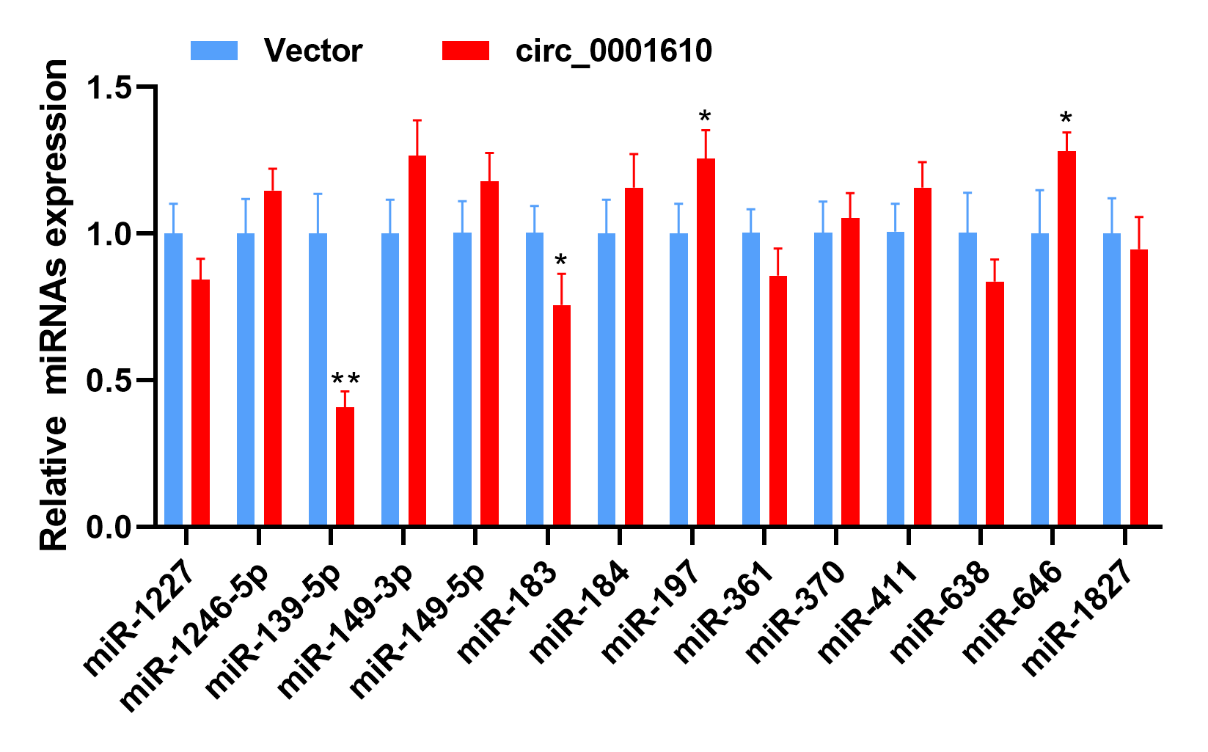
**Supplementary Figure 3** The expression levels of miRNAs in Ishikawa cells transfected with hsa_circ_0001610 or vector. **P*<0.05, ***P*<0.01 vs vector.
