# Supplementary material for: Exosomal transfer of tumor-associated macrophage-derived hsa_circ_0001610 reduces radiosensitivity in endometrial cancer
Source: Cell Death Dis. 2021 Aug 30;12(9):818. doi: 10.1038/s41419-021-04087-8 (PMC8405633; doi:10.1038/s41419-021-04087-8)
Supplement: Supplementary file 4 — Supplementary Figure 4 [file 41419_2021_4087_MOESM4_ESM.docx]

**
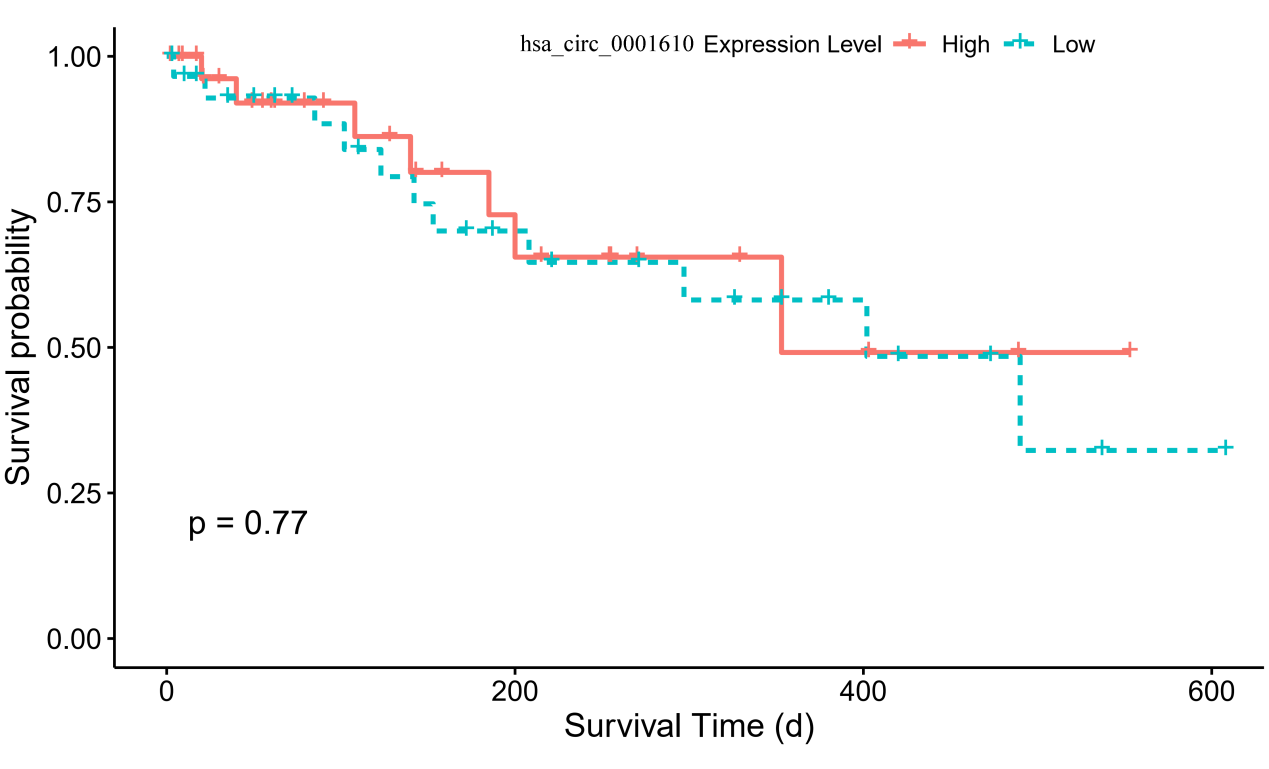
Supplementary Figure 4** The survival probability of EC patients with high hsa_circ_0001610 level or low hsa_circ_0001610 level (grouping based on the median expression).
